# Visual-physiological concordance and target vessel outcomes: a μQFR-based real-world cohort study

**DOI:** 10.3389/fcvm.2026.1785866

**Published:** 2026-03-20

**Authors:** Yanfeng Lu, Abdulrahman AlQazzaz, Jasmine Yimeng Bao, Gary S. Mintz, Jiahao Feng, Yong Zhang, Shanshan Gao, Qiang Song, Feifei Ning, Wangge Ma, Haoyu Wu, Yuejiao Wei, Jiaying Li, Ning Guo, Xin Huang

**Affiliations:** 1 Department of Cardiovascular Medicine, The First Affiliated Hospital of Xi’an Jiaotong University, Xi’an, Shaanxi, China; 2 Sidney Kimmel Medical College, Thomas Jefferson University, Philadelphia, PA, United States; 3 Cardiovascular Research Foundation, New York, NY, United States

**Keywords:** concordance, coronary artery disease, grey-zone, percutaneous coronary intervention, quantitative flow ratio, μQFR

## Abstract

**Background and objectives:**

Quantitative flow ratio (QFR) offers a non-interventional alternative for evaluating ischemia, yet its concordance with real-world treatment and clinical outcomes remains unclear. This study aimed to assess the alignment between Murray law-based QFR (μQFR) physiological assessment and treatment strategies, evaluate the association between treatment concordance and clinical outcomes, and explore factors influencing concordance.

**Methods:**

In this retrospective cohort, 242 patients with angiographically intermediate coronary stenosis and μQFR values adjacent to the grey-zone (low: 0.70–0.74; high: 0.86–0.90) were included. Treatment concordance was defined as PCI for low μQFR and medication for high μQFR. The primary endpoint was target vessel failure (TVF), comprising cardiac death, target vessel myocardial infarction, and ischemia-driven target vessel revascularization (ID-TVR). Median follow-up was 18 months. Kaplan–Meier analysis was used to evaluate outcomes, and logistic regression identified determinants of operator decision-making.

**Results:**

Among 242 patients, 177 (73.1%) received μQFR-concordant and 65 (26.9%) discordant treatment. TVF occurred less frequently in μQFR-concordant group (2.3% vs. 7.9%, *P* = 0.043), mainly due to reduced ID-TVR (1.1% vs. 6.4%, *P* = 0.024) and mainly driven by differences between the high- and low-μQFR medication subgroups. Operator decisions were influenced by lesion severity and overall coronary complexity, with more severe lesions favoring PCI and higher complexity favoring conservative management.

**Conclusions:**

Approximately one-fifth of treatment for intermediate coronary stenoses was discordant with μQFR assessment. μQFR-concordant treatment was associated with lower TVF, primarily due to fewer ID-TVR. Integrating μQFR into clinical decision-making may help optimize revascularization strategies and improve outcomes in real-world practice.

## 1 Introduction

Intermediate coronary stenosis remains an area of uncertainty in the clinical management of patients with coronary artery disease (CAD), as visual estimation of severity correlates poorly with ischemia. Both underestimation of ischemia resulting in inappropriate deferral of revascularization and unnecessary percutaneous coronary intervention (PCI) for physiologically non-significant lesions have been associated with an increased risk of adverse clinical events. While fractional flow reserve (FFR) provides a reliable physiological reference for revascularization decisions (1–6), its invasive nature and reliance on hyperemic agents limit its adoption. Quantitative flow ratio (QFR), a non-interventional technique that computes an FFR-equivalent value from one or two angiographic projections without pressure wires or hyperemia, has shown high concordance with FFR (7–11) and demonstrated clinical benefit in randomized trials (12, 13). However, most existing clinical evidence, including the FAVOR III trials, was generated using conventional 3D-QFR and dichotomous thresholds of 0.80 (14).

Evidence suggests uncertainty around this fixed cutoff. In FAVOR III China, outcomes for the 3D-QFR grey-zone subgroup were neutral (12, 15), whereas in FAVOR III Japan-Europe, 3D-QFR-guided revascularization strategies did not achieve non-inferiority compared with FFR guidance (16, 17). For vessels falling into QFR grey zone (0.75–0.85), analogous to the FFR grey zone (0.75–0.80) (18–20), neither conservative nor interventional strategies have demonstrated clear superiority, leaving therapeutic decisions ambiguous in clinical practice. Despite this, prior research has rarely examined the intervals adjacent to the grey zone, where uncertainty in visual assessment is also pronounced.

Leveraging the ability of QFR to be assessed retrospectively, our study excludes the grey zone itself and focuses on these adjacent intervals, where decision making is heterogeneous based on visual assessment. In this study, we used μQFR, a Murray-law-based QFR allowing single-projection computation, which has been shown to correlate well with FFR (11) while reducing angiographic requirements and enhancing the feasibility of retrospective assessment. We aim to evaluate the proportion of real-world clinical treatments that align with μQFR guidance, to examine the association between treatment concordance and clinical outcomes, and to explore factors associated with operator decision-making.

## 2 Materials and methods

### 2.1 Study design and population

**Figure 1:**
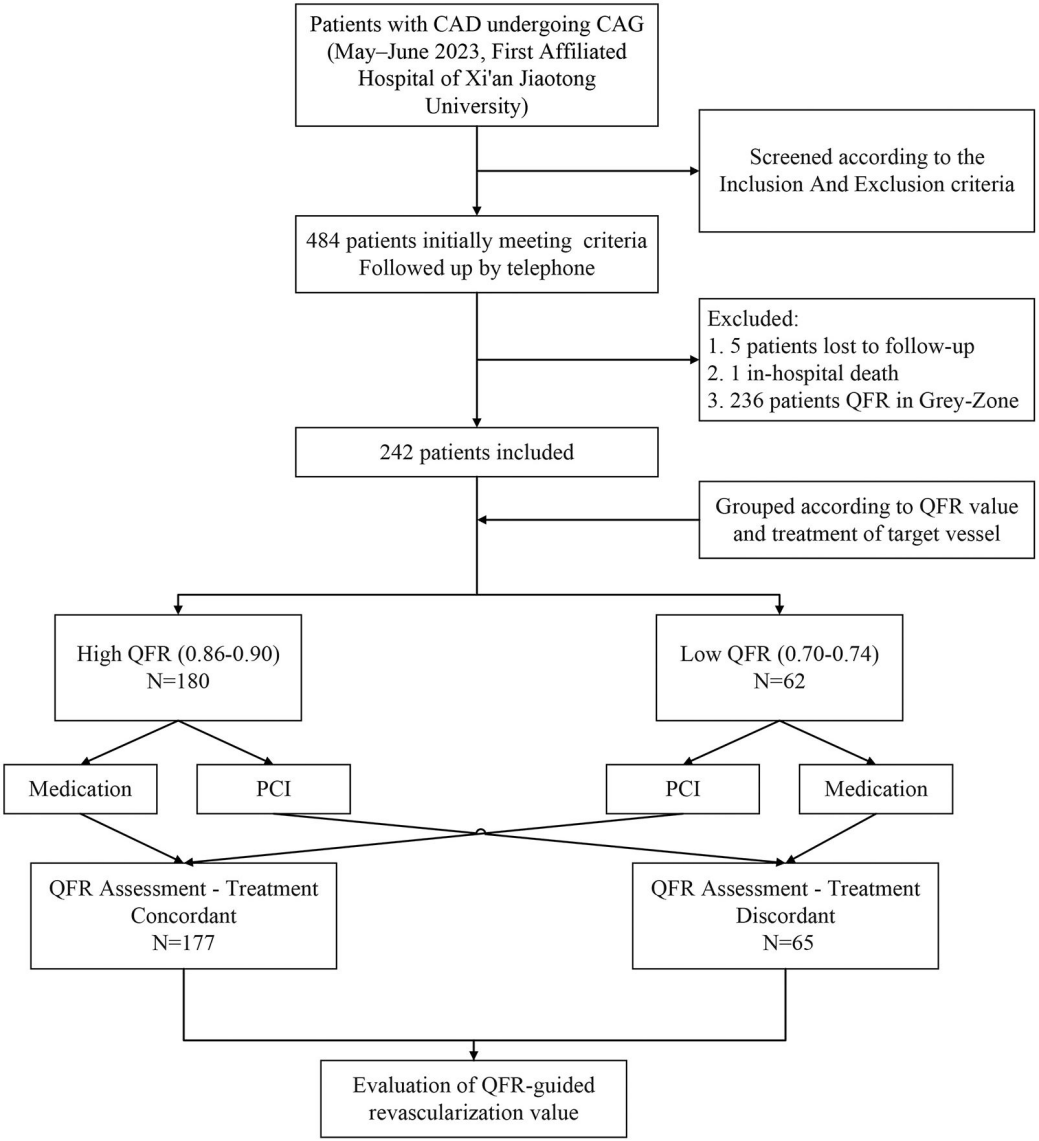
Enrollment flow chart.

This was a single-center, retrospective cohort study that included CAD patients undergoing coronary angiography at the First Affiliated Hospital of Xi'an Jiaotong University (Xi'an, China) between May and June 2023. Inclusion criteria were angiographic evidence of a single coronary artery with intermediate stenosis (diameter stenosis 30%–70% by visual assessment) and retrospective μQFR measurements between 0.70 and 0.90. Other coronary arteries were either free of significant disease or had undergone PCI for severe stenoses. Those with μQFR in the grey zone (0.75–0.85) were excluded due to uncertainty in treatment decision-making. Additional exclusion criteria included poor image quality, hemodynamic instability, >1 coronary artery with intermediate stenosis, left main or chronic total occlusion lesions, ostial lesions, stented target vessel, culprit lesions, in-hospital death, incomplete clinical data, or lost to/refusal of follow-up. The study flowchart is presented in Figure 1.

The studies involving humans were approved by the Clinical Research Ethics Committee of the First Affiliated Hospital, School of Medicine (SOM), Xi'an Jiaotong University. The studies were conducted in accordance with the local legislation and institutional requirements. The ethics committee/institutional review board waived the requirement of written informed consent for participation from the participants or the participants' legal guardians/next of kin because the retrospective nature of the study, using of de-identified patient records and posed no additional risk to participants.

### 2.2 Procedures and μQFR measurement

Coronary angiography and PCI were performed following standards of practices. μQFR was independently measured by two trained researchers following standardized procedures (11), and the mean value was recorded. In general, angiographic images were imported from the PACS system into the software (Angio Plus Gallery II, version 2.1; Shanghai Pulsed Medical Technology Co., Ltd., Shanghai, China), which automatically extracted main coronary artery and side branch lumen contours with calculations based on Murray's law. For eccentric lesions, a second angiographic projection was selected for calibration. Selection criteria prioritized minimal vessel overlap or foreshortening, clear visualization of the lesion lumen, adequate contrast filling, and complete dynamic sequence of vessel opacification. The software generated a structured report, including lesion length, minimum lumen diameter (MLD), and diameter stenosis (DS).

The SYNTAX Score I was independently calculated by trained interventional researchers, and categorized as low-risk (≤22), intermediate-risk (23–32), or high-risk (≥33) (21).

### 2.3 μQFR stratification and treatment concordance

Based on the definition of the 3D-QFR grey-zone adopted in FAVOR III China trial (12, 14), vessels with μQFR 0.75–0.85 were defined as the grey-zone. Accordingly, patients were initially grouped by target vessel μQFR: Low μQFR group (0.70–0.74), Grey zone μQFR group (0.75–0.85), and High μQFR group (0.86–0.90). In this study, vessels with μQFR <0.75 were considered physiologically significant, while those >0.85 were non-significant. Grey zone μQFR group was excluded from the primary analyses, due to uncertainty in treatment decision-making within this group.

Patients were then categorized according to the concordance between their actual treatment strategy and μQFR physiological assessment. The μQFR-concordant group (“Concordant”) included patients with low μQFR who underwent PCI and patients with high μQFR who received medical therapy. The μQFR-discordant group (“Discordant”) comprised patients who received treatment that contradicted μQFR guidance.

### 2.4 Data collection and endpoints

Baseline demographics, clinical history, angiographic characteristics, and treatment modalities (PCI vs. medication) were collected. The primary endpoint was target vessel failure (TVF), defined as a composite of cardiac death, non-fatal target vessel myocardial infarction (TV-MI), and ischemia-driven target vessel revascularization (ID-TVR). The secondary endpoint included major adverse cardiovascular events (MACE), a composite of cardiac death, non-fatal myocardial infarction (MI), and ischemia-driven revascularization (IDR); each individual component of TVF and MACE; and all-cause mortality. Unless an unequivocal non-cardiac cause was identified, any death of unknown cause was classified as cardiac death. Follow-up was conducted primarily by telephone, supplemented by review of electronic medical records when necessary. Investigators responsible for follow-up were blinded to μQFR results. All clinical events were independently adjudicated by two experienced cardiologists blinded to μQFR results; discrepancies were resolved by consensus or, if necessary, by consultation with a third senior cardiologist.

### 2.5 Statistical analysis

All analyses were performed using SPSS 27.0 and R 4.4.2. Continuous variables were expressed as mean ± standard deviation (SD) or median with interquartile range (IQR) and compared using Student's *t*-test or Mann–Whitney *U*-test, as appropriate. Categorical variables were expressed as counts and percentages and compared using the chi-square test or Fisher's exact test. Clinical events were analyzed using the Kaplan–Meier analysis with log-rank test, and expressed as the number of events and corresponding cumulative event rates. Sensitivity analyses were performed using Firth penalized Cox regression and leave-one-component-out Kaplan–Meier analysis to evaluate the robustness of the observed associations and to assess the contribution of each individual subgroup to the overall differences in event rates. Logistic regression was used to analyze the factors influencing decision-making.

Unless otherwise stated, all tests were two-sided with a significance level of *P* < 0.05. Bonferroni correction was applied for multiple comparisons.

## 3 Results

### 3.1 Baseline characteristics

A total of 1,993 patients undergoing coronary angiography were screened. Seventeen patients were excluded due to poor angiographic quality, including severe vessel overlap, foreshortening, or distortion, which prevented reliable μQFR calculation. 484 patients were preliminarily eligible with target vessel μQFR values between 0.70 and 0.90. Five patients were lost to follow-up and one patient died in-hospital, and 236 patients were excluded due to μQFR in the grey zone. Finally, 242 patients were included in the study. Overall, 73.1% (177/242) received treatment concordant to their physiological μQFR assessment; and 26.9% (65/242) received discordant treatment, including: 15.7% (38/242) who were over-treated (High μQFR + PCI) and 11.2% (27/242) who were under-treated (Low μQFR + Medication).

Table 1 presents the baseline clinical characteristics of patients grouped according to the concordance between physiological assessment and treatment strategy. No significant differences were observed between the Concordant and Discordant groups in baseline characteristics.

**Table 1:** Baseline clinical characteristics of patients grouped by concordance between µQFR physiological assessment and actual treatment strategy.

Clinical characteristics	Concordant	Discordant	*P* value
	(*N* = 177)	(*N* = 65)	
Age, years	65.0 (56.0, 71.0)	61.0 (53.5, 70.0)	0.336
<65	86 (48.6)	39 (60.0)	0.115
≥65	91 (51.4)	26 (40.0)	
Sex (*n*, %)			0.555
Male	121 (68.4)	47 (72.3)	
Female	56 (31.6)	18 (27.7)	
BMI, kg/m^2^	24.22 (22.31, 26.57)	24.77 (22.71, 27.39)	0.531
<30	166 (93.8)	64 (98.5)	0.137
≥30	11 (6.2)	1 (1.5)	
Diabetes mellitus (*n*, %)	70 (39.5)	24 (36.9)	0.710
Hypertension (*n*, %)	106 (59.9)	36 (55.4)	0.528
Hyperlipidemia (*n*, %)	60 (33.9)	20 (30.8)	0.646
History of Statins (*n*, %)	97 (54.8)	35 (53.8)	0.895
Smoking (*n*, %)			0.127
Never	80 (45.2)	22 (33.8)	
Former	21 (11.9)	9 (13.8)	
Active	76 (42.9)	34 (52.3)	
Prior MI (*n*, %)	25 (14.1)	16 (24.6)	0.054
Prior PCI (*n*, %)	55 (31.1)	18 (27.7)	0.611
LDL-C, mmol/L	1.87 (1.42, 2.60)	1.83 (1.38, 2.38)	0.283
HbA1c, %	6.1 (5.8, 6.8)	6.1 (5.8, 6.7)	0.772
eGFR, mL/min/1.73 m^2^			0.414
≥60	167 (94.4)	63 (96.9)	
<60	10 (5.6)	2 (3.1)	
LVEF (*n*, %)			0.925
>45	156 (88.1)	57(87.7)	
≤45	21(11.9)	8(12.3)	

Baseline angiographic characteristics such as minimal lumen diameter (MLD) and diameter stenosis (DS) differed between the concordant and discordant groups, primarily due to a higher proportion of low μQFR group in the discordant group (Table 2). Other angiographic characteristics were generally comparable (Table 2).

**Table 2:** Angiographic characteristics of patients grouped by concordance between μQFR physiological assessment and actual treatment strategy.

Angiographic Characteristics	Concordant	Discordant	*P* value
	(*N* = 177)	(*N* = 65)	
Length, mm	23.57 (14.79, 36.21)	23.98 (14.80, 35.38)	0.956
MLD, mm	1.74 (1.48, 2.19)	1.59 (1.32, 1.96)	0.011
DS, %	41.7 (36.9, 47.6)	46.2 (40.8, 52.4)	<0.001
µQFR Group (*n*, %)			<0.001
High	142 (80.2)	38 (58.5)	
Low	35 (19.8)	27 (41.5)	
No. of diseased vessels			0.311
1 (*n*, %)	31 (17.5)	17 (26.2)	
2 (*n*, %)	71 (40.1)	22 (33.8)	
3 (*n*, %)	75 (42.4)	26 (40.0)	
Calcification (*n*, %)	53 (29.9)	21 (32.3)	0.723
Tandem lesion (*n*, %)	34 (19.2)	15 (23.1)	0.507
SYNTAX Score Ⅰ	20.0 (12.0, 30.0)	24.0 (12.5, 33.2)	0.397
Low (≤22)	101 (57.1)	33 (50.8)	0.435
Intermediate (23–32)	43 (24.3)	15 (23.1)	
High (≥33)	33(18.6)	17(26.2)	

### 3.2 Clinical outcomes

**Figure 2:**
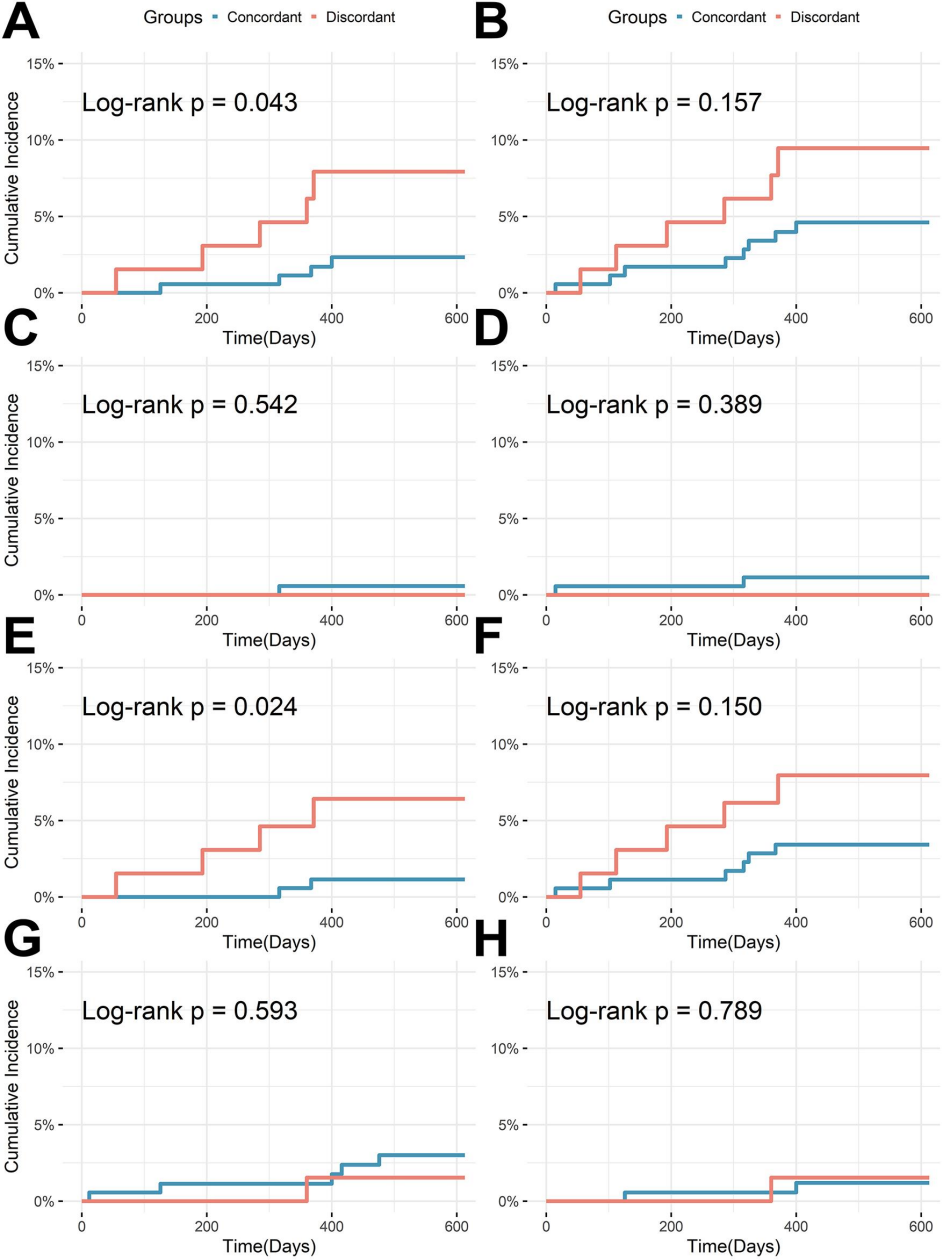
Kaplan–meier curves for clinical outcomes according to concordant status. **(A)** Target vessel failure (TVF); **(B)** Major adverse cardiovascular events (MACE); **(C)** Target vessel myocardial infarction (TV-MI); **(D)** Myocardial infarction (MI); **(E)** Ischemia-driven target vessel revascularization (ID-TVR); **(F)** Ischemia-driven revascularization (IDR); **(G)** All-cause mortality; **(H)** Cardiac death.

During the median 18-month follow-up, a total of 9 patients experienced TVF and 13 experienced MACE (Table 3). For the primary endpoint TVF, the discordant group had a significantly higher cumulative incidence compared to the concordant group (7.9% vs. 2.3%, Log-rank *P* = 0.043), primarily driven by a significantly higher incidence of ID-TVR in the discordant group (6.4% vs. 1.1%, Log-rank *P* = 0.024). Although the cumulative incidence of MACE was lower in the concordant vs. the discordant group (4.6% vs. 9.5%), the difference did not reach statistical significance (Log-rank *P* = 0.157; Table 3 and Figure 2).

**Table 3:** Clinical outcomes by μQFR-concordant and discordant.

Clinical Outcomes	Concordant	Discordant	*P* value
	(*N* = 177)	(*N* = 65)	
Follow-up, days	553 (496, 574)	559 (523, 584)	0.559
All-cause mortality	5 (3.0)	1 (1.5)	0.593
TVF	4 (2.3)	5 (7.9)	0.043
Cardiac death	2 (1.2)	1 (1.5)	0.789
TV-MI	1 (0.6)	0 (0)	0.542
ID-TVR	2 (1.1)	4 (6.4)	0.024
MACE	8 (4.6)	6 (9.5)	0.157
Cardiac death	2 (1.2)	1 (1.5)	0.789
MI	2 (1.1)	0 (0)	0.389
IDR	6 (3.4)	5 (8.0)	0.150

**Figure 3:**
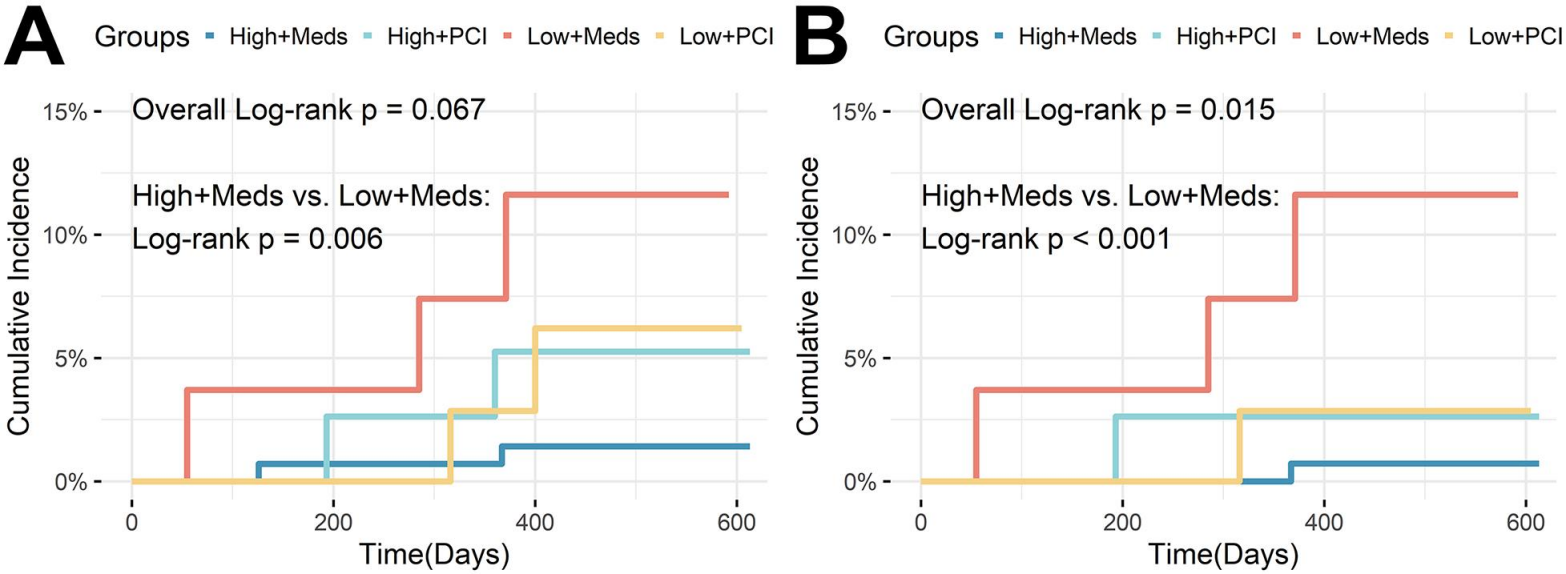
Kaplan–meier curves for μQFR-treatment subgroups. **(A)** Target vessel failure (TVF); **(B)** Ischemia-driven target vessel revascularization (ID-TVR).

Event rates differed across the four subgroups (Figure 3). The High μQFR + Medication group had a significantly lower cumulative incidence of TVF compared with the Low μQFR + Medication group (1.4% vs. 11.6%, Log-rank *P* = 0.006) primarily attributed to ID-TVR (0.7% vs. 11.6%, Log-rank *P* < 0.001).

### 3.3 Sensitivity analyses

Sensitivity analysis using univariable Firth penalized Cox regression showed that patients in the discordant group were 3.56 times and 5.09 times more likely to experience TVF (HR = 3.484, 95% CI 0.988–12.961, *P* = 0.052) and ID-TVR (HR = 5.092, 95% CI 1.129–29.209, *P* = 0.034) than those in the concordant group. While the confidence interval is excessively wide and the association with TVF did not reach the predefined significance threshold, likely due to the small number of TVF (*n* = 9) and ID-TVR (*n* = 6) events, the direction and magnitude of the risk were consistent with the Kaplan–Meier survival analysis.

Leave-one-out analysis was performed using Kaplan–Meier analysis to assess whether the observed between-group difference was driven by specific μQFR-treatment subgroups. Overall, the exclusion of any single μQFR-treatment subgroup did not materially alter the overall trend for TVF and ID-TVR, and the association between μQFR-treatment concordance and lower incidence of adverse events remained stable (Supplementary Tables S1, S2; Figures S1, S2). However, excluding the High μQFR + Medication or Low μQFR + Medication subgroups, rendered the association non-significant. Conversely, excluding the High μQFR + PCI or Low μQFR + PCI subgroups strengthened the association.

In sensitivity analyses using a conventional cutoff of 0.80, μQFR-concordant treatment remained significantly associated with a lower risk of TVF and ID-TVR, with effect estimates consistent in direction with the primary analysis (Supplementary Table S11).

### 3.4 Determinants of treatment discordance

**Figure 4:**
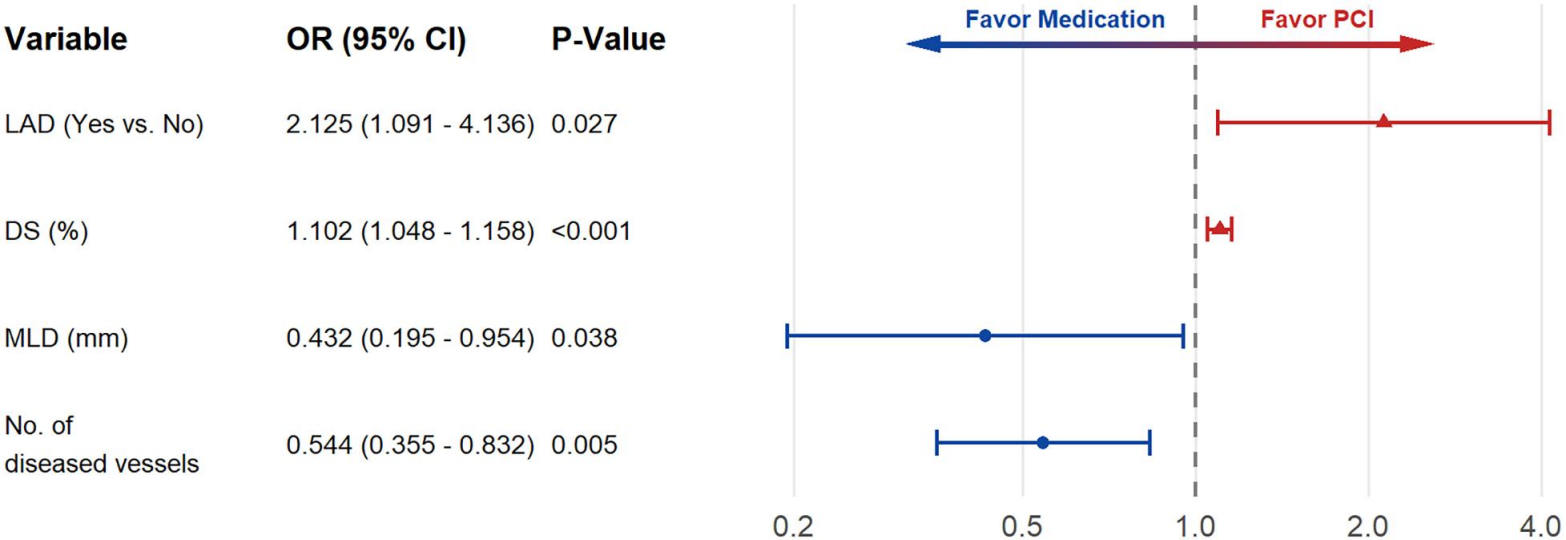
Multivariable logistic regression analysis of factors associated with performing PCI. DS, diameter stenosis; MLD, minimal lumen diameter.

In this cohort, 30.2% (73/242) of the target vessels underwent PCI. To explore operators' decision-making preferences, logistic regression analyses were performed using PCI (yes/no) as the dependent variable. Several angiographic variables were associated with the operator's decision to perform PCI in univariable analysis (Supplementary Table S3). Stenosis in the LAD, with smaller minimal lumen diameter (MLD), higher diameter stenosis (DS), and fewer diseased vessels were more likely to undergo PCI. Variables with *P* < 0.10 were then entered into the multivariable model using the forward likelihood ratio (LR) method. In multivariable logistic regression, these four factors remained independently associated with performing PCI (Figure 4, Supplementary Table S4).

Within each μQFR Group, determinants of treatment discordance were further assessed. Among patients with high μQFR, discordant treatment (PCI despite physiologically non-significant ischemia) occurred in 21.1% (38/180). Compared with concordant cases (medication), discordant cases had shorter lesion length, smaller MLD, higher DS and fewer diseased vessels (Supplementary Tables S5, S6). Multivariable logistic regression confirmed MLD (adjusted OR = 0.283, 95% CI: 0.119–0.677, *P* = 0.005) and the numbers of diseased vessels (adjusted OR = 0.529, 95% CI: 0.329–0.849, *P* = 0.008) as independent factors (Supplementary Table S7).

Among patients with low μQFR, discordant cases (medication despite physiologically significant ischemia) accounted for 43.5% (27/62). Compared with concordant cases (PCI), discordant cases showed longer lesion length and higher anatomical complexity (SYNTAX group) (Supplementary Tables S8, S9). In the final multivariable logistic regression model, only SYNTAX group was retained (OR = 2.252, 95% CI: 1.170–4.337, *P* = 0.015).

## 4 Discussion

In this retrospective cohort analysis based on a real-world clinical population guided by conventional angiography, we analyzed patients with CAD who were identified as having single-vessel intermediate stenosis, with a specific focus on the μQFR ranges adjacent to the grey zone (μQFR 0.70–0.74 and 0.86–0.90), which represent clinically ambiguous territories where decision-making is most uncertain, to investigate the impact and determinants of μQFR–angiography treatment concordance. The main findings were as follows: (1) μQFR and angiography-guided treatment decisions were discordant in approximately 26.9% of the patients, underscoring the substantial inconsistency between visual assessment and physiological severity. (2) Treatment strategies concordant with physiologic significance based on μQFR was associated with a reduced risk of target vessel failure (TVF), mainly driven by a lower incidence of target vessel revascularization. (3) Operator decisions were primarily influenced by angiographic severity, vessel-level severity (smaller MLD, higher DS) favored PCI decisions, while greater overall anatomical complexity (multivessel disease, higher SYNTAX score) tended to conservative management.

Quantitative flow ratio (QFR), derived directly from angiographic images, provides a wire- and hyperemia-free alternative to FFR for identifying ischemia. In our real-world cohort, 73.1% of patients received treatment strategies aligned with physiological assessment, while approximately 26.9% were treated discordantly, including both overtreatment (15.7%) and undertreatment (11.2%). This proportion differs significantly from that reported in previous similar studies (22–24), possibly because prior studies used a binary threshold of 0.80, while our analysis excluded the grey zone and was based on routine clinical practice. Similarly, in the FAVOR III China Trial, physiological assessment altered the initial treatment strategy in 23.3% of patients (12). These findings underscore the limitations of angiography alone in identifying ischemia and highlight the clinical value of physiological assessment in providing objective guidance.

Consistent with previous studies based on 3D-QFR (22, 23, 25, 26), our results demonstrated that patients receiving treatment concordant to µQFR physiological assessment were associated with a reduced risk of adverse events, primarily attributed to the reduced risk of ID-TVR. Notably, subgroup and sensitivity analyses suggest that this difference is mainly driven by the clinical outcomes between patients in the high-μQFR group who received concordant medication therapy and those in the low μQFR group who received conservative treatment. This finding is consistent with the findings of Zhang et al. (22), in which the increased risk observed in the discordant group was primarily attributed to insufficient revascularization of ischemic vessels. However, Terentes-Printzios et al. (23) and Lee et al. (26) demonstrated that excessive PCI in vessels with high 3D-QFR values was also associated with an increased risk in the discordant group. This discrepancy may be partly explained by the different thresholds adopted, as our study excluded the grey zone where the optimal treatment strategy remains uncertain. Moreover, the adverse effects of unnecessary revascularization on non-significant ischemia vessels appear to be time-dependent. For instance, the DEFER trial demonstrated that a higher incidence of myocardial infarction associated with over-intervention emerged only after long-term follow-up of 15 years (4, 6). Given the relatively small sample size and limited follow-up in our study, the statistical power may have been insufficient to capture such long-term differences. Therefore, larger prospective studies with extended follow-up are still needed to confirm the impact of overtreatment.

Our analysis also offers insight into operator preferences in real-world clinical practice. At the vessel level, revascularization decisions were influenced by anatomical severity, as smaller minimal lumen diameter and greater diameter stenosis independently increased the likelihood of PCI, with also a tendency to intervene stenosis in LAD. In contrast, greater overall coronary complexity, particularly in patients with multivessel disease, was associated with a preference for conservative management of target vessels. These findings indicate that operators may be discouraged from intervening in intermediate stenoses when procedural complexity is high and may instead prioritize more severe lesions. Since MLD, DS and lesion length are closely inherently related, we performed an exploratory principal component analysis (PCA) to summarize them and capture their combined “visual severity.” This composite severity dimension (PC1) remained strongly associated with PCI decisions (*P* < 0.001, Supplementary Table S10, Figure S3, S4), reflecting an overall visual impression guiding operators rather than reliance on a single parameter.

These patterns became more distinct in physiological subgroups. In high µQFR group, overtreatment was associated with visually severe but functionally mild stenoses, whereas in low µQFR group undertreatment tended to occur in diffuse or complex disease where ischemia may be underestimated and the degree of focal narrowing at the vessel level was not the primary determinant of operator decision-making. Our findings suggest that operator decision-making is shaped by two competing cognitive mechanisms: visual overestimation of focal severity and avoidance of intervening in diffusely diseased or complex anatomy, together explaining the occurrence of both overtreatment and undertreatment. Importantly, these decisions, particularly in the low µQFR group, may not necessarily represent “errors,” but rather reflect reasonable clinical judgments that balance perceived anatomical risk, procedural complexity, and long-term outcomes based on angiographic information alone, rather than focal lesion underestimation *per se*. Consistent with previous studies (1, 24, 27), these findings highlight that angiography-based revascularization decisions are still guided by visual assessment and anatomical features, which can conflict with physiological severity. In this context, µQFR provided physiological clarity, helping correct systematic biases inherent in angiography-guided decision-making.

Unlike previous study (22, 23, 28), we specifically focus on the µQFR ranges adjacent to the grey zone, and patients within the µQFR grey zone (0.75–0.85) were excluded to minimize misclassification and strengthen the validity of concordance analyses. The FAVOR II Europe-Japan Study has demonstrated that the agreement between 3D-QFR and FFR is highest at physiological extremes, but declines substantially within the grey zone due to measurement variability (9). Furthermore, grey zone subgroup analyses from trials such as FAVOR III China have shown nonsignificant clinical benefits (12, 15). By excluding this range, our study ensured clearer separation between physiologically significant and non-significant lesions, allowing more reliable evaluation of concordant vs. discordant treatment strategies. Notably, in our cohort, nearly half of the patients initially eligible for analysis (236 of 478) had µQFR grey zone values, underscoring that this subgroup represents a substantial proportion of real-world intermediate lesions and remains an area of ongoing clinical uncertainty.

### 4.1 Limitations

This study has several limitations. First, it is a single-center study, and the proportion of μQFR-concordant vs. discordant treatment observed may reflect practices specific to our institution, limiting generalizability to other centers. Second, as a retrospective study, it is subject to inherent biases and potential confounding. Although sensitivity analyses were performed, unmeasured factors such as operator experience, treatment preferences and patients' subjective conditions may still influence the results. Therefore, the underlying rationale for discordant treatment decisions and its outcomes cannot be fully disentangled. Third, the sample size and follow-up duration were limited, reducing statistical power and limiting assessment of infrequent events such as cardiac death, myocardial infarction, and the impact of overtreatment. Fourth, MLD and DS were measured by software rather than by operators' visual assessment, and do not directly represent the subjective visual judgment. Finally, the exclusion of patients with μQFR values in the grey zone limits applicability to this clinically important subgroup. Additionally, differences between μQFR and conventional 3D-QFR assessments may limit the comparability of our results with studies using 3D-QFR, warranting cautious interpretation.

## 5 Conclusion

About one-fifth of treatment decisions for intermediate coronary stenoses was discordant with µQFR guidance. µQFR-concordant management was associated with fewer target vessel events, primarily by reducing ischemia-driven revascularization. Integrating µQFR into clinical decision-making may help optimize revascularization strategies and improve clinical outcomes in real-world practice.

## Data Availability

The raw data supporting the conclusions of this article will be made available by the authors, without undue reservation.
