# Supplementary material for: Visual-physiological concordance and target vessel outcomes: a μQFR-based real-world cohort study
Source: Front Cardiovasc Med. 2026 Mar 20;13:1785866. doi: 10.3389/fcvm.2026.1785866 (PMC13047079; doi:10.3389/fcvm.2026.1785866)
Supplement: Supplementary file 1 [file Datasheet1.docx]

### Supplementary material:

#### Leave-one-component-out Sensitivity Analysis

To assess the robustness of the association between μQFR-treatment concordance and outcomes and whether the observed between-group difference was driven by specific μQFR-treatment subgroups, a leave-one-component-out analysis was performed using Kaplan–Meier analysis. When each of the four subgroups (high μQFR + medication, high μQFR + PCI, low μQFR + PCI, low μQFR + medication) was sequentially excluded, the trend toward lower TVF and ID-TVR rates in the concordant group was preserved overall. The statistical significance weakened when either the high μQFR + medication or low μQFR + medication subgroup was removed, whereas exclusion of the high μQFR + PCI or low μQFR + PCI subgroup enhanced the significance (Supplementary Tables S1 and S2; Supplementary Figures S1 and S2).

Table S1 Leave-one-component-out sensitivity analysis for TVF.

| **Excluded subgroup** | **Remaining subgroups** | | **P value** | **Direction of effect** |
| --- | --- | --- | --- | --- |
|  | Concordant | Discordant |  |  |
| High μQFR + Medication | Low μQFR + PCI | High μQFR + PCI, Low μQFR + Medication | 0.691 | Consistent |
| High μQFR + PCI | High μQFR + Medication, Low μQFR + PCI | Low μQFR + Medication | 0.015 | Consistent |
| Low μQFR + PCI | High μQFR + Medication | High μQFR + PCI, Low μQFR + Medication | 0.019 | Consistent |
| Low μQFR + Medication | High μQFR + Medication, Low μQFR + PCI | High μQFR + PCI | 0.300 | Consistent |


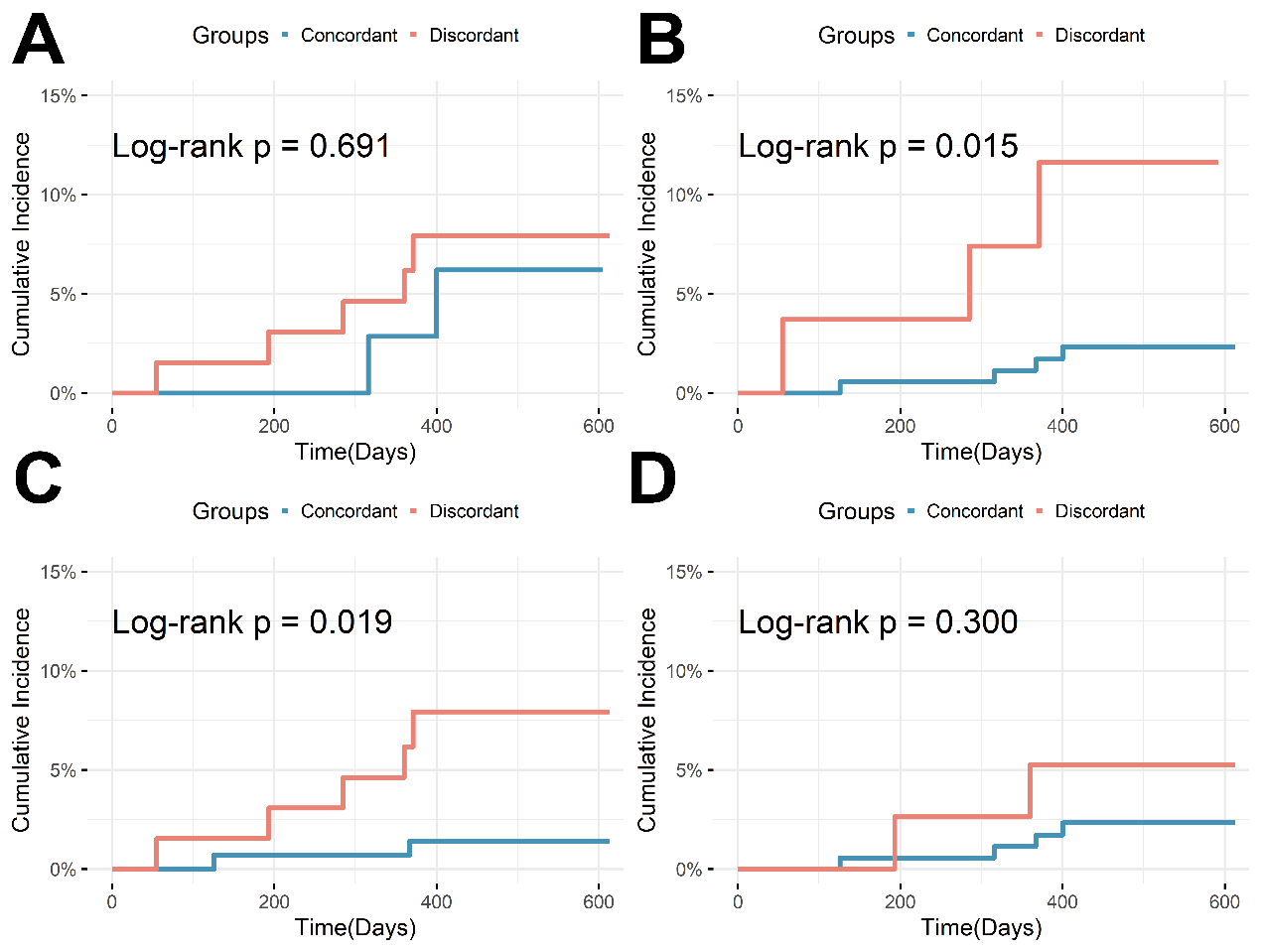


Figure S1 Kaplan–Meier curves for TVF after sequential exclusion of each μQFR-treatment subgroup. A: exclusion of High μQFR + Medication; B: exclusion of High μQFR + PCI; C: exclusion of Low μQFR + PCI; D: exclusion of Low μQFR + Medication.

Table S2. Leave-one-component-out sensitivity analysis for ID-TVR.

| **Excluded subgroup** | **Remaining subgroups** | | **P value** | **Direction of effect** |
| --- | --- | --- | --- | --- |
|  | Concordant | Discordant |  |  |
| High μQFR + Medication | Low μQFR + PCI | High μQFR + PCI, Low μQFR + Medication | 0.465 | Consistent |
| High μQFR + PCI | High μQFR + Medication, Low μQFR + PCI | Low μQFR + Medication | 0.002 | Consistent |
| Low μQFR + PCI | High μQFR + medication | High μQFR + PCI, Low μQFR + Medication | 0.016 | Consistent |
| Low μQFR + Medication | High μQFR + Medication, Low μQFR + PCI | High μQFR + PCI | 0.466 | Consistent |


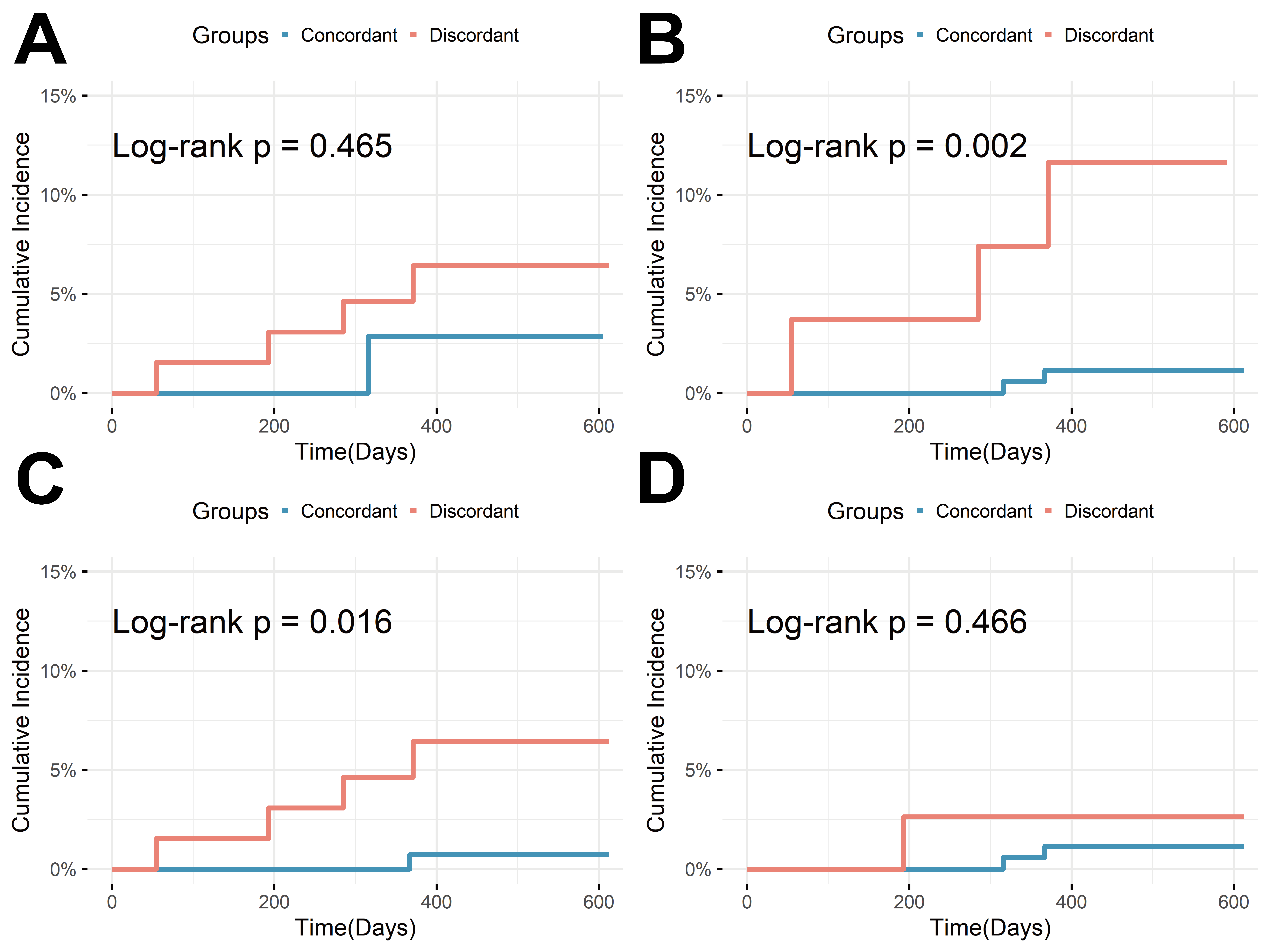


Figure S2 Kaplan–Meier curves for ID-TVR after sequential exclusion of each μQFR-treatment subgroup. A: exclusion of High μQFR + Medication; B: exclusion of High μQFR + PCI; C: exclusion of Low μQFR + PCI; D: exclusion of Low μQFR + Medication.

#### Logistic Regression for factors associated with performing PCI

Table S3 Univariable logistic regression for factors associated with performing PCI in the overall cohort.

| **Variable (Ref)** | **OR** | **95% CI** | | **P value** |
| --- | --- | --- | --- | --- |
| **Age (<65)** | 0.835 | 0.481 | 1.448 | 0.521 |
| **Sex (Female)** | 1.244 | 0.678 | 2.285 | 0.481 |
| **BMI, kg/m2** | 1.072 | 0.983 | 1.169 | 0.117 |
| **Diabetes mellitus (No)** | 0.822 | 0.465 | 1.452 | 0.499 |
| **Hypertension (No)** | 0.735 | 0.422 | 1.279 | 0.276 |
| **Hyperlipidemia (No)** | 0.623 | 0.339 | 1.146 | 0.128 |
| **Smoking (No)** | 1.060 | 0.790 | 1.422 | 0.699 |
| **Prior PCI (No)** | 0.998 | 0.549 | 1.815 | 0.995 |
| **Prior MI (No)** | 1.422 | 0.703 | 2.879 | 0.327 |
| **LDL-C, mmol/L** | 0.835 | 0.593 | 1.176 | 0.303 |
| **HbA1c, %** | 0.930 | 0.731 | 1.183 | 0.556 |
| **eGFR Group (≥60 ml/min/1.73m2)** | 1.167 | 0.340 | 4.003 | 0.806 |
| **LVEF (>45%)** | 0.709 | 0.288 | 1.741 | 0.453 |
| **Target Vessel (non-LAD)** | 1.748 | 1.010 | 3.045 | 0.046 |
| **Length, mm** | 0.988 | 0.970 | 1.007 | 0.208 |
| **MLD, mm** | 0.202 | 0.102 | 0.399 | <0.001 |
| **DS, %** | 1.106 | 1.064 | 1.149 | <0.001 |
| **No. of diseased vessels (1)** | 0.619 | 0.427 | 0.890 | 0.010 |
| **Calcification (No)** | 1.064 | 0.588 | 1.926 | 0.837 |
| **Tandem lesion (No)** | 1.300 | 0.668 | 2.528 | 0.440 |
| **SYNTAX Group (Low Risk)** | 0.784 | 0.549 | 1.120 | 0.181 |
|  | | | | |

Variables with P<0.10 were then entered into the multivariable logistic regression model using the forward likelihood ratio (LR) method.

Table S4 Multivariable logistic regression for factors associated with performing PCI.

| **Variable (Ref)** | **OR** | **95% CI** | | **P value** |
| --- | --- | --- | --- | --- |
| **Target Vessel (non-LAD)** | 2.125 | 1.091 | 4.136 | 0.027 |
| **MLD, mm** | 0.432 | 0.195 | 0.954 | 0.038 |
| **DS, %** | 1.102 | 1.048 | 1.158 | <0.001 |
| **No. of diseased vessels (1)** | 0.544 | 0.355 | 0.832 | 0.005 |
| No multicollinearity was detected among the included variables, based on collinearity diagnostics (0 < VIF < 5 and Tolerance > 0.1). | | | | |

**Determinants of treatment discordance in High μQFR Group**

Table S5 Clinical and angiographic characteristics of patients grouped by concordance between μQFR physiological assessment and actual treatment strategy in High μQFR Group.

| **Clinical characteristics** | **Concordant** | **Discordant** | **P value** |
| --- | --- | --- | --- |
|  | (N=142) | (N=38) |  |
| **Age, years** | 65.0(55.8, 71.0) | 61.0(50.0, 69.0) | 0.313 |
| **<65** | 70(49.3) | 24(63.2) | 0.129 |
| **≥65** | 72(50.7) | 14(36.8) |  |
| **Sex (n, %)** |  |  | 0.627 |
| **Male** | 95(66.9) | 27(71.1) |  |
| **Female** | 47(33.1) | 11(28.9) |  |
| **BMI, kg/m^2^** | 24.14(22.22, 26.67) | 25.66(24.22, 27.55) | 0.053 |
| **<30** | 133(93.7) | 37(97.4) | 0.691 |
| **≥30** | 9(6.3) | 1(2.6) |  |
| **Diabetes mellitus (n, %)** | 55(38.7) | 11(28.9) | 0.266 |
| **Hypertension (n, %)** | 85(59.9) | 18(47.4) | 0.167 |
| **Hyperlipidemia (n, %)** | 52(36.6) | 11(28.9) | 0.378 |
| **History of Statins (n, %)** | 77(54.2) | 14(36.8) | 0.057 |
| **Smoking (n, %)** |  |  |  |
| **Never** | 65(45.8) | 14(36.8) | 0.301 |
| **Former** | 15(10.6) | 4(10.5) |  |
| **Active** | 62(43.7) | 20(52.6) |  |
| **Prior MI (n, %)** | 18(12.7) | 8(21.1) | 0.192 |
| **Prior PCI (n, %)** | 40(28.2) | 7(18.4) | 0.224 |
| **LDL-C, mmol/L** | 1.91(1.44, 2.62) | 1.97(1.47, 2.43) | 0.744 |
| **HbA1c, %** | 6.1(5.8, 6.8) | 6.0(5.8, 6.6) | 0.302 |
| **eGFR, ml/min/1.73m2** |  |  | 1.000 |
| **≥60** | 135(95.1) | 37(97.4) |  |
| **<60** | 7(4.9) | 1(2.6) |  |
| **LVEF (n, %)** |  |  | 0.371 |
| **>45** | 126(88.7) | 36(94.7) |  |
| **≤45** | 16(11.3) | 2(5.3) |  |
|  |  |  |  |
| **Length, mm** | 21.60(13.99, 33.61) | 15.95(11.73, 24.64) | 0.030 |
| **MLD, mm** | 1.92(1.59, 2.27) | 1.67(1.38, 2.00) | 0.005 |
| **DS, %** | 39.7(35.9, 44.1) | 43.0(38.9, 47.4) | 0.008 |
| **No. of diseased vessels** |  |  | 0.009 |
| **1 (n, %)** | 26(18.3) | 15(39.5) |  |
| **2 (n, %)** | 52(36.6) | 12(31.6) |  |
| **3 (n, %)** | 64(45.1) | 11(28.9) |  |
| **Calcification (n, %)** | 37(26.1) | 7(18.4) | 0.331 |
| **Tandem lesion (n, %)** | 23(16.2) | 6(15.8) | 0.952 |
| **SYNTAX Score Ⅰ** | 20.0(11.8, 30.0) | 16.0(7.0, 28.0) | 0.167 |
| **Low (≤22)** | 82(57.7) | 27(71.1) | 0.265 |
| **Intermediate (23**–**32)** | 34(23.9) | 5(13.2) |  |
| **High (≥33)** | 26(18.3) | 6(15.8) |  |

Table S6 Univariable logistic regression for factors associated with discordance in High μQFR Group.

| **Variable (Ref)** | **OR** | **95% CI** | | **P value** |
| --- | --- | --- | --- | --- |
| **Age (<65)** | 0.567 | 0.271 | 1.185 | 0.131 |
| **Sex (Female)** | 1.214 | 0.555 | 2.658 | 0.627 |
| **BMI, kg/m2** | 1.075 | 0.963 | 1.201 | 0.197 |
| **Diabetes mellitus (No)** | 0.644 | 0.296 | 1.403 | 0.268 |
| **Hypertension (No)** | 0.607 | 0.296 | 1.237 | 0.169 |
| **Hyperlipidemia (No)** | 0.705 | 0.323 | 1.538 | 0.380 |
| **Smoking (No)** | 1.224 | 0.834 | 1.794 | 0.301 |
| **Prior PCI (No)** | 0.576 | 0.235 | 1.413 | 0.228 |
| **Prior MI (No)** | 1.837 | 0.730 | 4.625 | 0.197 |
| **LDL-C, mmol/L** | 0.865 | 0.557 | 1.344 | 0.519 |
| **HbA1c, %** | 0.808 | 0.554 | 1.179 | 0.268 |
| **eGFR Group (≥60 ml/min/1.73m2)** | 0.521 | 0.062 | 4.371 | 0.548 |
| **LVEF (>45%)** | 0.438 | 0.096 | 1.992 | 0.285 |
| **Target Vessel (non-LAD)** | 1.563 | 0.762 | 3.208 | 0.223 |
| **Length, mm** | 0.961 | 0.931 | 0.993 | 0.016 |
| **MLD, mm** | 0.292 | 0.123 | 0.694 | 0.005 |
| **DS, %** | 1.082 | 1.019 | 1.150 | 0.010 |
| **No. of diseased vessels (1)** | 0.545 | 0.342 | 0.866 | 0.010 |
| **Calcification (No)** | 0.641 | 0.260 | 1.579 | 0.333 |
| **Tandem lesion (No)** | 0.970 | 0.364 | 2.584 | 0.952 |
| **SYNTAX Group (Low Risk)** | 0.755 | 0.459 | 1.240 | 0.266 |
|  | | | | |

Variables with P<0.10 were then entered into the multivariable logistic regression model using the forward likelihood ratio (LR) method.

Table S7 Multivariable logistic regression for factors associated with performing PCI.

| **Variable (Ref)** | **OR** | **95% CI** | | **P value** |
| --- | --- | --- | --- | --- |
| **MLD, mm** | 0.283 | 0.119 | 0.677 | 0.005 |
| **No. of diseased vessels (1)** | 0.529 | 0.329 | 0.849 | 0.008 |
| No multicollinearity was detected among the included variables, based on collinearity diagnostics (0 < VIF < 5 and Tolerance > 0.1). | | | | |

#### Determinants of treatment discordance in Low μQFR Group

Table S8 Clinical and angiographic characteristics of patients grouped by concordance between μQFR physiological assessment and actual treatment strategy in Low μQFR Group.

| **Clinical characteristics** | **Concordant** | **Discordant** | **P value** |
| --- | --- | --- | --- |
|  | (N=35) | (N=27) |  |
| **Age, years** | 67.0(58.0, 71.0) | 61.5(55.8, 73.3) | 0.473 |
| **<65** | 16(45.7) | 15(55.6) | 0.442 |
| **≥65** | 19(54.3) | 12(44.4) |  |
| **Sex (n, %)** |  |  | 0.985 |
| **Male** | 26(74.3) | 7(25.9) |  |
| **Female** | 9(25.7) | 20(74.1) |  |
| **BMI, kg/m^2^** | 24.57(22.73, 26.05) | 23.28(21.73, 25.80) | 0.117 |
| **<30** | 33(94.3) | 27(100) | 0.500 |
| **≥30** | 2(5.7) | 0(0) |  |
| **Diabetes mellitus (n, %)** | 15(42.9) | 13(48.1) | 0.678 |
| **Hypertension (n, %)** | 21(60.0) | 18(66.7) | 0.590 |
| **Hyperlipidemia (n, %)** | 8(22.9) | 9(33.3) | 0.359 |
| **History of Statins (n, %)** | 20(57.1) | 21(77.8) | 0.110 |
| **Smoking (n, %)** |  |  |  |
| **Never** | 15(42.9) | 8(29.6) | 0.282 |
| **Former** | 6(17.1) | 5(18.5) |  |
| **Active** | 14(40.0) | 14(51.9) |  |
| **Prior MI (n, %)** | 7(20.0) | 8(29.6) | 0.380 |
| **Prior PCI (n, %)** | 15(42.9) | 11(40.7) | 0.867 |
| **LDL-C, mmol/L** | 1.80(1.30, 2.36) | 1.62(1.25, 2.31) | 0.523 |
| **HbA1c, %** | 6.2(5.8, 6.9) | 6.3(6.0, 6.9) | 0.788 |
| **eGFR, ml/min/1.73m2** |  |  | 0.626 |
| **≥60** | 32(91.4) | 26(96.3) |  |
| **<60** | 3(8.6) | 1(3.7) |  |
| **LVEF (n, %)** |  |  | 0.510 |
| **>45** | 30(85.7) | 21(77.8) |  |
| **≤45** | 5(14.3) | 6(22.2) |  |
|  |  |  |  |
| **Length, mm** | 31.66±12.48 | 40.13±18.45 | 0.035 |
| **MLD, mm** | 1.37±0.31 | 1.52±0.51 | 0.193 |
| **DS, %** | 53.3±6.69 | 51.0±6.80 | 0.177 |
| **No. of diseased vessels** |  |  | 0.070 |
| **1 (n, %)** | 5(14.3) | 2(7.4) |  |
| **2 (n, %)** | 19(54.3) | 10(37.0) |  |
| **3 (n, %)** | 11(31.4) | 15(55.6) |  |
| **Calcification (n, %)** | 16(45.7) | 14(51.9) | 0.632 |
| **Tandem lesion (n, %)** | 11(31.4) | 9(33.3) | 0.874 |
| **SYNTAX Score Ⅰ** | 23.2±11.6 | 31.8±13.6 | 0.009 |
| **Low (≤22)** | 19(54.3) | 6(22.2) | 0.013 |
| **Intermediate (23**–**32)** | 9(25.7) | 10(37.0) |  |
| **High (≥33)** | 7(20.0) | 11(40.7) |  |

Table S9 Univariable logistic regression for factors associated with discordance in Low μQFR Group.

| **Variable (Ref)** | **OR** | **95% CI** | | **P value** |
| --- | --- | --- | --- | --- |
| **Age (<65)** | 0.674 | 0.246 | 1.848 | 0.443 |
| **Sex (Female)** | 0.989 | 0.314 | 3.114 | 0.985 |
| **BMI, kg/m2** | 0.867 | 0.719 | 1.044 | 0.132 |
| **Diabetes mellitus (No)** | 1.238 | 0.451 | 3.396 | 0.678 |
| **Hypertension (No)** | 1.333 | 0.468 | 3.801 | 0.590 |
| **Hyperlipidemia (No)** | 1.688 | 0.549 | 5.191 | 0.361 |
| **Smoking (No)** | 1.365 | 0.776 | 2.399 | 0.280 |
| **Prior PCI (No)** | 0.917 | 0.331 | 2.539 | 0.867 |
| **Prior MI (No)** | 1.684 | 0.523 | 5.425 | 0.382 |
| **LDL-C, mmol/L** | 0.958 | 0.502 | 1.830 | 0.897 |
| **HbA1c, %** | 1.038 | 0.697 | 1.547 | 0.853 |
| **eGFR Group (≥60 ml/min/1.73m2)** | 0.410 | 0.040 | 4.181 | 0.452 |
| **LVEF (>45%)** | 1.714 | 0.462 | 6.362 | 0.420 |
| **Target Vessel (non-LAD)** | 0.441 | 0.158 | 1.234 | 0.119 |
| **Length, mm** | 1.037 | 1.001 | 1.074 | 0.042 |
| **MLD, mm** | 2.523 | 0.685 | 9.292 | 0.164 |
| **DS, %** | 0.948 | 0.877 | 1.025 | 0.178 |
| **No. of diseased vessels (1)** | 2.112 | 0.932 | 4.787 | 0.073 |
| **Calcification (No)** | 1.279 | 0.468 | 3.497 | 0.632 |
| **Tandem lesion (No)** | 1.091 | 0.373 | 3.187 | 0.874 |
| **SYNTAX Group (Low Risk)** | 2.252 | 1.170 | 4.337 | 0.015 |
|  | | | | |

Variables with a P<0.10 in the univariable analysis were entered into the model using the forward likelihood ratio (LR) method, including length, number of diseased vessels and SYNTAX group. No multicollinearity was detected among these included variables, based on collinearity diagnostics (0 < VIF < 5 and Tolerance > 0.1). In the final model, only SYNTAX risk group was retained.

#### Principal Component Analysis and Logistic Regression

A principal component analysis (PCA) was performed to summarize the anatomical characteristics of the target vessel. Three parameters were included: minimal lumen diameter (MLD), percent diameter stenosis (DS), and lesion length. All variables were standardized before analysis.

PCA identified three components explaining 52.8%, 33.9%, and 13.4% of the total variance, respectively (Table S12). PC1 primarily represents a pattern of lesion severity: lower PC1 values correspond to smaller minimal lumen diameter and higher diameter stenosis, indicating more severe narrowing. Lesion length has a modest positive loading (0.226), suggesting that lesions with lower PC1 tend to be slightly shorter and more localized, but stenosis severity remains the dominant feature. PC2 largely represented lesion length (loading = 0.936), while PC3 contributed minimal independent variance (13.4%) and was not used for further modeling (Table S12). To facilitate regression modeling, PC1 scores were standardized (PC1_z), with lower values indicating more severe stenosis.

Table S10 PCA of angiographic variables (MLD, DS, Length) with variance and loadings.

|  | **PC1** | **PC2** | **PC3** |
| --- | --- | --- | --- |
| **Variable** | 2.125 | 1.091 | 4.136 |
| **MLD, mm** | 0.712 | 0.032 | 0.702 |
| **DS, %** | -0.665 | 0.352 | 0.659 |
| **Length, mm** | 0.226 | 0.936 | -0.272 |
| **Proportion of Variance** | 0.528 | 0.339 | 0.133 |
| **Cumulative Proportion** | 0.528 | 0.867 | 1.000 |


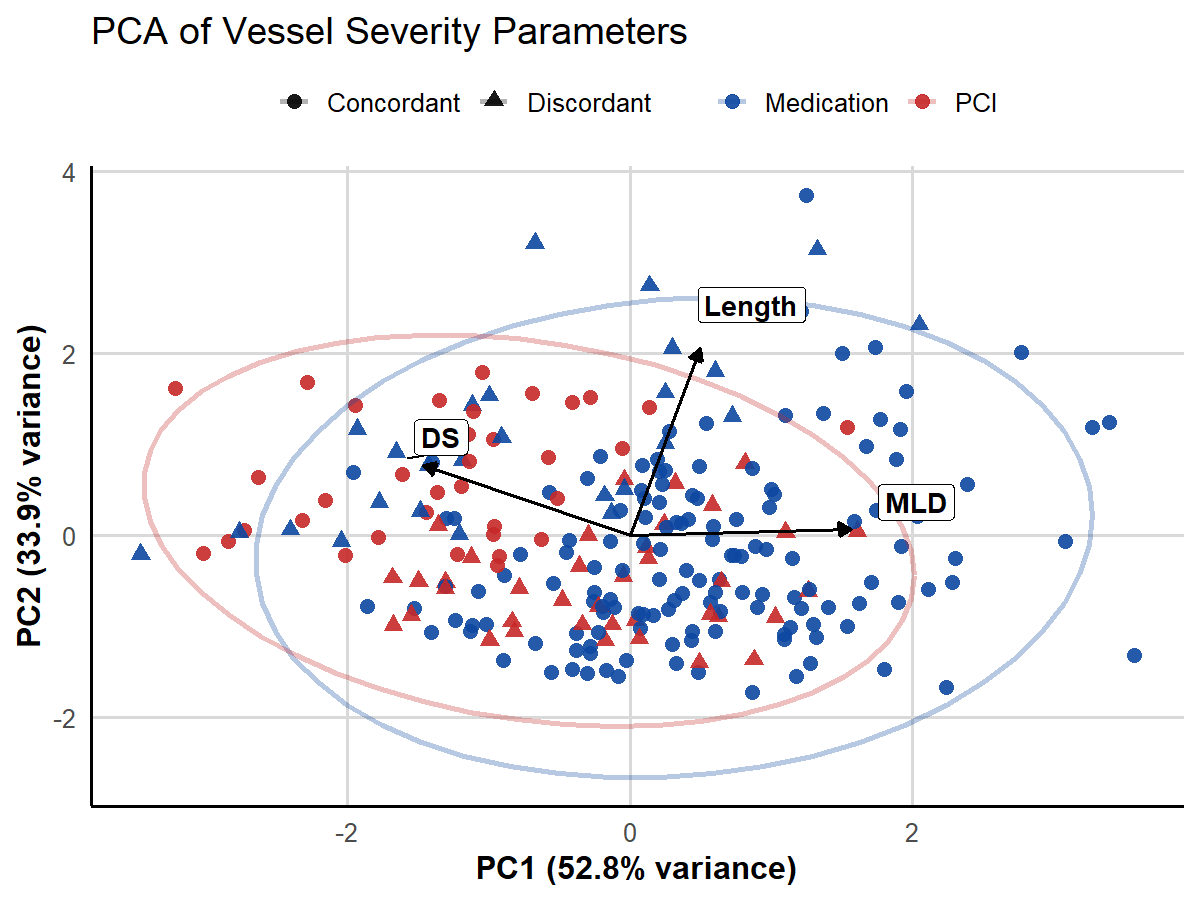


Figure S3 Principal Component Analysis of Vessel Severity Parameters.


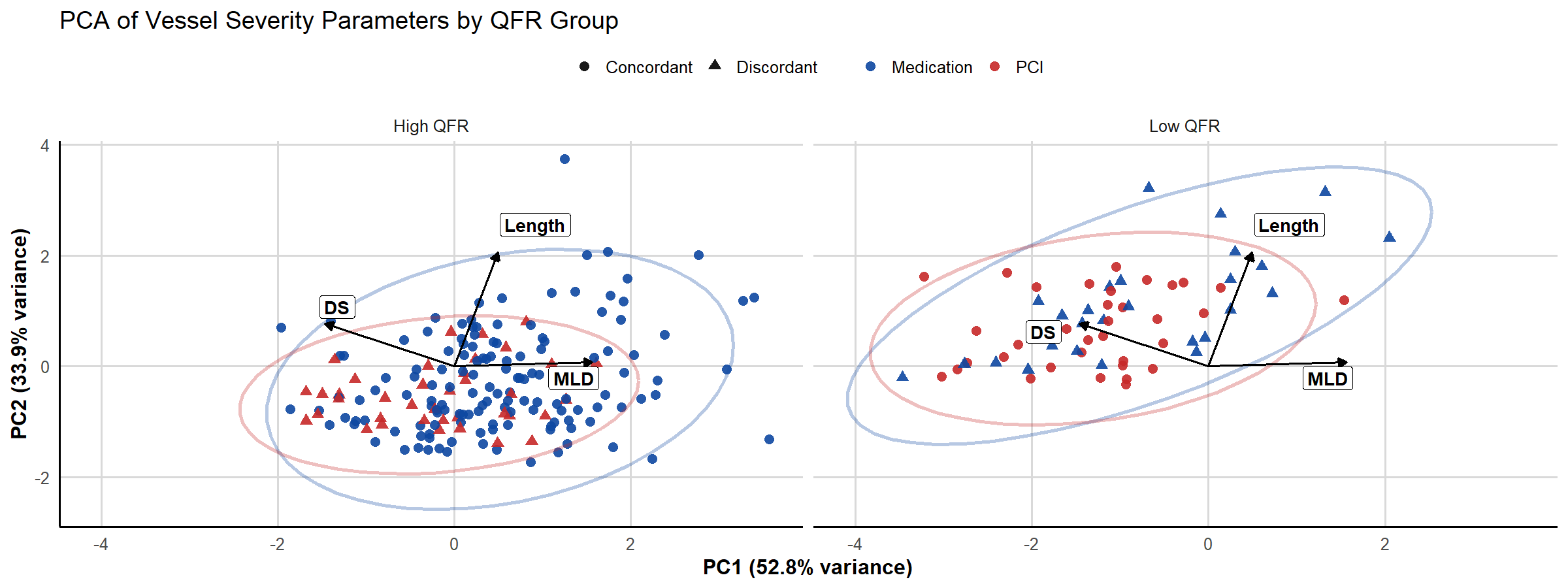


Figure S4 Principal Component Analysis of Vessel Severity Parameters Stratified by High and Low μQFR Group.

To explore the impact of anatomical severity on operator decisions, logistic regression with Firth penalization was applied.

In the overall cohort (n=242), PC1_z was strongly associated with PCI (OR=0.389, 95% CI 0.271–0.541, P<0.001). After adjusting for target vessel in LAD and number of diseased vessels, PC1_z remained an independent predictor of performing PCI (adjusted OR=0.345, 95% CI 0.233–0.492, P<0.001). These findings suggest that operators tended to intervene on more severe, localized lesions.

In High μQFR Group, PC1_z remained an independent determinant of overtreatment (PCI despite non-significant ischemia). PC1_z was inversely associated with performing PCI (adjusted OR=0.406, 95% CI 0.237–0.661, P<0.001), indicating a persistent reliance on visual stenosis severity though physiology non-significant. Additionally, fewer diseased vessels favored PCI (adjusted OR=0.515, 95% CI 0.315–0.824, P=0.006), implying that operators may have been more inclined to treat an isolated visually severe lesion.

In Low μQFR Group, PC1_z showed only a marginal association (OR=1.662, 95% CI 0.951–3.105, P=0.075) with undertreatment (medication despite significant ischemia), as well as PC2_z (OR=1.828, 95% CI 0.991–3.638, P=0.054). Instead, SYNTAX group was the main driver of discordance (OR=2.178, 95% CI 1.172–4.258, P=0.013). This suggests that undertreatment in the Low μQFR group was primarily influenced by overall coronary complexity than by focal stenosis severity, in which operators may have avoided PCI despite physiological ischemia.

#### Sensitivity analyses using 0.80 as a cut-off

In a sensitivity analysis, treatment concordance was alternatively defined using a conventional cutoff of 0.80. Vessels with μQFR ≤0.80 were considered physiologically significant, and those with μQFR >0.80 were considered non-significant.

μQFR-treatment concordance was defined using the same framework as in the primary analysis: patients with μQFR ≤0.80 treated with PCI and those with μQFR >0.80 treated with medical therapy were classified as concordant, while all other treatment patterns were classified as discordant.

Using a conventional μQFR cutoff of 0.80, μQFR-treatment concordance remained significantly associated with reduced adverse outcomes. Compared with the concordant group, patients receiving μQFR-discordant treatment had a significantly higher risk of target vessel failure (TVF, 6.6% vs. 2.3%, Log-rank P=0.026) and ischemia-driven target vessel revascularization (ID-TVR, 6.0% vs. 1.3%, Log-rank P=0.006).

In Firth penalized Cox regression analysis, μQFR-discordant treatment was associated with a 2.80-fold increased risk of TVF (HR=2.80, 95% CI 1.11–7.46, P=0.030) and a 4.16-fold increased risk of ID-TVR (HR=4.16, 95% CI 1.24–17.13, P=0.021).

These findings were directionally consistent with the primary analysis, supporting the robustness of the association between μQFR-treatment concordance and clinical outcomes.

Table S11 Clinical Outcomes by μQFR-concordant and discordant using a μQFR cutoff of 0.80

| **Clinical Outcomes** | **Concordant** | **Discordant** | **P value** |
| --- | --- | --- | --- |
|  | (N=314) | (N=164) |  |
| **TVF** | 7 (2.3) | 10 (6.6) | 0.026 |
| **Cardiac death** | 3 (1.0) | 1 (0.6) | 0.712 |
| **TV-MI** | 2 (0.6) | 2 (1.5) | 0.508 |
| **ID-TVR** | 4 (1.3) | 9 (6.0) | 0.006 |
